# *Cancer Imaging* reviewer acknowledgement 2015

**DOI:** 10.1186/s40644-016-0060-x

**Published:** 2016-02-03

**Authors:** Rodney J. Hicks

**Affiliations:** Cancer Imaging, The Peter MacCallum Cancer Centre, East Melbourne, Australia; The Sir Peter MacCallum Department of Oncology, University of Melbourne Parkville, Melbourne, Australia

## Abstract

The Editor of *Cancer Imaging* would like to thank all reviewers who have contributed their time and expertise to the journal in Volume 15 (2015).

**Nicolas Aide**

France

**Gopinathan Anil**

United Kingdom

**Mine Araz**

Turkey

**Alexandra Athanasiou**

Greece

**Anil Attili**

United States

**Ravikanth Balaji**

India

**Sebastiano Barbieri**

Switzerland

**Ahmed Ba-Ssalamah**

Austria

**Alberto Bazzocchi**

Italy

**Guy Burkill**

United Kingdom

**John Buscombe**

United Kingdom

**Meltem Caglar**

Turkey

**Ammar Chaudhry**

United States

**Chan Yoon Cheah**

Australia

**Arturo Chiti**

Italy

**Emmanuel Christodoulou**

United States

**Conor Collins**

Ireland

**Clemens Cyran**

Germany

**Frederik De Keyzer**

Belgium

**Roberto Delgado Bolton**

United Kingdom

**Stefan Diederich**

Germany

**Michel Eisenblaetter**

United Kingdom

**Olgun Elicin**

Switzerland

**Andrew Evans**

United Kingdom

**Jacob Farnebo**

Sweden

**Rosemarie Forstner**

Austria

**Isaac Francis**

United States

**Julia Furtner**

Austria

**Maryam Ghadimi Mahani**

United States

**Francesco Giammarile**

France

**Emma Goldstraw**

United Kingdom

**Nelly Gordillo**

Mexico

**Chiara Grana**

Italy

**Sonja Greenslade**

Australia

**Lubomir Hadjiiski**

United States

**Matthias Hammon**

Germany

**Chris Harvey**

United Kingdom

**Alexander Haug**

Austria

**Rodney Hicks**

Australia

**Michael Ingrisch**

Germany

**Amir Iravani**

Australia

**Shingo Iwano**

Japan

**Avinash Kambadakone**

United States

**Raghava Kashyap**

India

**Linges Kasilingam**

Malaysia

**Ann King**

China

**Kimberly Kirschner**

United States

**Bernd Klaeser**

Switzerland

**Dow-Mu Koh**

United Kingdom

**Hisanobu Koyama**

Japan

**Yulia Lakhman**

United States

**Heng Liu**

China

**Iain Lyburn**

United Kingdom

**Bill Majdalany**

United States

**Luis Marti-Bonmati**

Spain

**Anthony Maxwell**

United Kingdom

**Beth Mccarville**

United States

**Kieran Mchugh**

United Kingdom

**Ken Miles**

United Kingdom

**Sophia Mueller**

Germany

**Iqbal Munir**

Saudi Arabia

**Dominik Nörenberg**

Germany

**Nour-Eldin**

Germany

**Elizabeth O'Flynn**

United Kingdom

**Lars J. Petersen**

Denmark

**Helmut Prosch**

Austria

**Andrea Rockall**

United Kingdom

**J. P. Ruurda**

Netherlands

**Anju Sahdev**

United Kingdom

**Evis Sala**

United States

**Wolfgang Schima**

Austria

**Heinz-Peter Schlemmer**

Germany

**Moritz Schneider**

Germany

**Mario Silva**

Italy

**Aslam Sohaib**

United Kingdom

**Ravi Srinivasa**

United States

**Sylvia Tomas**

United Kingdom

**Vincent Vandecaveye**

Belgium

**Sobhan Vinjamuri**

United Kingdom

**Dennis Vriens**

Netherlands

**David Wang**

United States

**Ashish Wasnik**

United Kingdom

**Yoichi Watanabe**

United States

**Shelley Waugh**

United Kingdom

**Roland Wiest**

Switzerland

**Jonathon Willatt**

United States

**Geoffrey Zhang**

United States

